# A simultaneous EEG-fMRI study of thalamic load-dependent working memory delay period activity

**DOI:** 10.3389/fnbeh.2023.1132061

**Published:** 2023-02-23

**Authors:** Bernard A. Gomes, Chelsea Reichert Plaska, Jefferson Ortega, Timothy M. Ellmore

**Affiliations:** ^1^Program in Cognitive Neuroscience, The Graduate Center of the City University of New York, New York, NY, United States; ^2^Doctoral Program in Behavioral and Cognitive Neuroscience, The Graduate Center of the City University of New York, New York, NY, United States; ^3^Department of Psychology, The City College of the City University of New York, New York, NY, United States

**Keywords:** thalamus, delay activity, working memory, simultaneous EEG-fMRI, source estimation

## Abstract

**Introduction:**

Working memory (WM) is an essential component of executive functions which depend on maintaining task-related information online for brief periods in both the presence and absence of interfering stimuli. Active maintenance occurs during the WM delay period, the time between stimulus encoding and subsequent retrieval. Previous studies have extensively documented prefrontal and posterior parietal cortex activity during the WM delay period, but the role of subcortical structures including the thalamus remains to be fully elucidated, especially in humans.

**Methods:**

Using a simultaneous electroencephalogram (EEG)-functional magnetic resonance imaging (fMRI) approach, we investigated the role of the thalamus during the WM delay period in a modified Sternberg paradigm following low and high memory load encoding of naturalistic scenes. During the delay, participants passively viewed scrambled scenes containing similar color and spatial frequency to serve as a perceptual baseline. Individual source estimation was weighted by the location of the thalamic fMRI signal relative to the WM delay period onset.

**Results:**

The effects memory load on maintenance were observed bilaterally in thalamus with higher EEG source amplitudes in the low compared to high load condition occurring 160–390 ms after the onset of the delay period.

**Conclusion:**

The main finding that thalamic activation was elevated during the low compared to high condition despite similar duration of perceptual input and upcoming motor requirements suggests a capacity-limited role for sensory filtering of the thalamus during consolidation of stimuli into WM, where the highest activity occurs when fewer stimuli need to be maintained in the presence of interfering perceptual stimuli during the delay. The results are discussed in the context of theories regarding the role of the thalamus in sensory gating during working memory.

## 1. Introduction

Working memory (WM) is the ability to maintain and manipulate information for the guidance of goal-directed behavior (Baddeley, 2003; Logie et al., 2020). WM encompasses processing functions including the active maintenance of stimuli for goal-related planning (Baddeley and Hitch, 1974). Maintenance occurs during the WM delay period, the time after stimulus encoding but before retrieval or recognition. Much research on WM is devoted to understanding the neural systems and neural changes during the delay period that support WM.

Early studies by Fuster and Alexander (1971) and others in non-human primates revealed that neurons in the prefrontal cortex (PFC) show elevated levels of action potential firing during the maintenance phase of delayed-response tasks (Funahashi et al., 1989). This neural signature is thought to represent the temporary memory storage of the stimulus in WM (Fuster and Alexander, 1971). Fuster and Alexander (1971) first reported that changes were observed during the delay period during a short-term memory task in the PFC and in the mediodorsal nucleus of the thalamus (MDt). Research has suggested that the neural activity generated during the delay period is maintained in the cortex, particularly the anterior lateral motor (ALM) cortex (Courtney et al., 1997; Inagaki et al., 2019). However, this idea has been challenged recently, including a study that found that the maintenance of information is dependent on delay activity in the thalamus (Guo et al., 2017).

Although the long-standing view of the thalamus is that it serves as a relay station for all major sensory pathways, it has more recently been suggested that thalamus plays a role in memory and cognition by maintaining and updating relevant information (Wolff and Vann, 2019). Electrophysiological recordings have suggested that specifically MDt is involved in WM (Sommer and Wurtz, 2006; Mair et al., 2015). MDt sends connections to PFC, a region implicated in WM maintenance (Mitchell, 2015). MDt also receives inputs from parahippocampal regions and is also reciprocally connected to the medial prefrontal cortex (mPFC) (Jang and Yeo, 2014; Yang et al., 2019). The role for MDt in WM may be to facilitate persistent activity in the PFC (McCormick and Bal, 1994; Yang et al., 2019). MDt and the anterior thalamus have also been implicated in familiarity and recollection, respectively (Kafkas et al., 2020), where it was also reported that ventral posteromedial and pulvinar thalamic nuclei regions were involved in scene familiarity with greater activity for familiar scenes compared to new scenes. More recently discovery of a gene encoding an orphan G-protein-coupled receptor, Gpr12, found that it enables high thalamus-PFC synchrony to support memory maintenance and choice accuracy (Hsiao et al., 2020).

Taken together these results supports an emerging thalamus-centric framework that supplements classical PFC-based models for a mechanistic understanding of WM. Several questions remain, however, including the nature of the thalamic response after encoding complex stimuli as well as during the presentation of interfering stimuli during the WM delay period. Critically, thalamic relays to and from dorsolateral prefrontal cortex (dlPFC) have been suggested to support suppression of interfering environmental stimuli during the delay (Postle, 2005). During the delay period, the thalamus may regulate sensory processing by up- or down-regulating potentially disruptive sensory information (Knight et al., 1999). The ability to suppress sensory input is likely also load-dependent. WM load has been extensively studied to understand if there are limits in how much information can be maintained in WM and how the brain can maintain multiple items (Fukuda et al., 2010). These studies have found that during the delay period, the dlPFC and the middle and superior frontal gyri show increased activity as load increases. In contrast, other areas show the opposite relationship including left caudal inferior frontal gyrus which shows increased activity as WM load decreases (Manoach et al., 1997; Rypma et al., 1999, 2002).

The objective of the present study is to characterize the load-dependent response of the thalamus and specifically Mdt during a complex visual WM task while participants are presented with interfering stimuli during the delay period. If the role of Mdt is to facilitate WM, then one hypothesis is that as load increases so should delay activity to support the higher demands on maintenance. An alternative hypothesis is that that the ability of Mdt to suppress sensory input during WM maintenance is load-dependent. This leads to the prediction that at higher WM loads, the thalamus response will be reduced reflecting an inability to filter interfering stimuli when there are high demands on maintenance. When WM load is lower, the thalamus response will be higher, reflecting an increased ability to filter disruptive sensory information because demands on maintenance are lower. We tested these predictions using the simultaneous functional magnetic resonance imaging (fMRI)-electroencephalogram (EEG) technique, which allows for quantifying hemodynamic changes at relatively high spatial resolution and using this spatial information to weight the estimation of higher temporal resolution EEG sources.

## 2. Materials and methods

### 2.1. Participants

This simultaneous EEG-fMRI experiment was conducted under a protocol approved by the Institutional Review Board of The City University of New York Human Research Protection Program (CUNY HRPP IRB). All methods were carried out in accordance with the relevant guidelines and regulations of the CUNY HRPP IRB committee. All participants (12 males, 12 females, mean age 25.3 years, SD = 8.5, and age range 18–54) were recruited either by flyers posted throughout the City College of New York campus or by web postings on the City College of New York SONA online experimental scheduling system. All participants had normal or corrected-to-normal vision with no reported neurological or psychiatric disorders. Each participant provided written informed consent and completed study procedures according to a protocol approved by the Institutional Review Board of the Human Research Protection Program. Participants were either compensated $15 per h or received one psychology course extra credit per hour of participation in the study.

### 2.2. Task, Stimuli, and experimental paradigm

**FIGURE 1:**
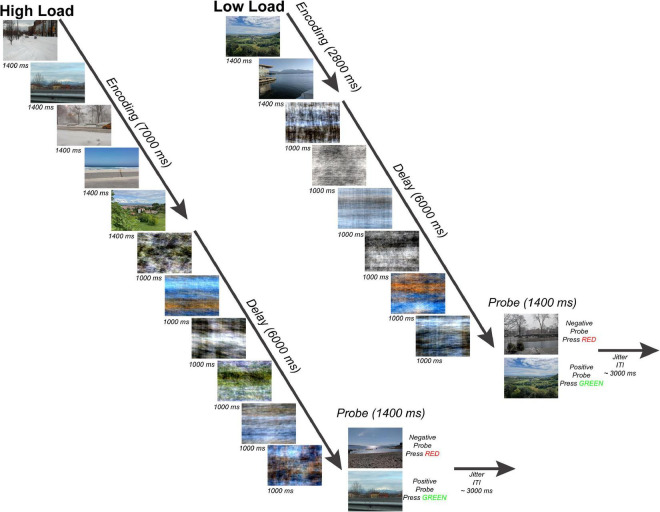
Working Memory Task. Subjects performed a modified Sternberg task with naturalistic scenes consisting of 50 trials per working memory load. There were two working memory loads: high load-5 images **(left)** and low load-2 images **(right)**. Subjects viewed either 5 or 2 sequentially presented images (encoding phase), maintained the scenes across a 6-s length delay period (maintenance phase), and after determined whether a probe scene matched one of the previous images seen during that trial or was a non-match (probe phase). Each trial ended with a jittered inter-trial fixation interval (mean = 3 s).

Participants completed a variant of a Sternberg WM task (Sternberg, 1966) with scenes as stimuli (Figure 1). The task consisted of an encoding period (two or five scenes presented for 1,400 ms each for a total 7,000 ms encoding period), a delay period (six phase-scrambled scenes each presented for 1,000 ms for a delay period duration of 6,000 ms), and a probe choice (a single scene presented for 1,400 ms with 50% probability of matching one of the previous scenes presented at encoding), and an end-of-trial jitter period (average 3,000 ms). The scenes presented during the encoding period were randomly selected from the SUN database (Xiao et al., 2010) and consisted of a set of 671 800-by-600 pixel novel color outdoor scenes which were presented on a BOLDscreen LCD monitor (Cambridge Research Systems, Rochester, UK) behind the scanner and viewed by each participant through a mirror mounted above the head coil. The Fourier phase-scrambled scenes presented during the delay period served as a perceptual baseline allowing for a comparison of activity during WM encoding with activity during WM maintenance with visual input during the delay period consisting of similar color and spatial frequency information. This paradigm was motivated by the results of a separate pilot behavioral experiment in which participants saw either a standard fixation cross or phase-scrambled scenes during the delay period (Supplementary Figure 1).

During the single EEG-fMRI session, each participant completed 50 trials of the low-load WM task and 50 trials of the high-load WM task presented in separated runs with order counterbalanced and randomized. Before each session, participants were instructed to do the task by presentation of three example trials containing stimuli different from the experimental runs. The experimenter read task instructions from a script for all participants during the practice trials outside the scanner and before each experimental run inside the scanner. No instruction was given to participants about when to blink during the task. Blink artifacts were confirmed by visual inspection of EEG data later during processing and removed from the EEG data before ERP averaging and source analysis. The probe responses were coded as a digitized button press signal sent via fiber optic cable to a USB interface located (fORP 932, Current Designs, Ltd., Philadelphia, USA) in the MRI scanner control room.

### 2.3. EEG Acquisition, Preprocessing, and ERP generation

Electroencephalogram data were sampled at 2.5 kHz using a BrainAmp-MR system (Brain Products GmbH, Gilching, Germany) placed behind the participant’s head inside the MRI scanner. Participants were fitted with a 32-channel MR-compatible BrainCap-MR cap (Brain Products GmbH, Gilching, Germany) containing a 10-10 system montage with 31 scalp electrodes (Fp1, Fp2, F7, F3, Fz, F4, F8, FC5, FC1, FC2, FC6, T7, C3, Cz, C4, T8, CP5, CP1, CP2, CP6, TP9, TP10, P7, P3, Pz, P4, P8, POz, O1, Oz, and O2) and one electrode for recording ballistocardiogram (BCG) placed on the left shoulder blade. Electrode impedances were initially lowered to below 20 kOhm and were monitored to keep them below 50 kOhm during recording according to Brain Products safety guidelines. All electrode signals were referenced to Fpz during acquisition and then all scalp electrodes were re-referenced to the common average reference offline after the experiment. EEG data were analyzed with Brain Electrical Source Analysis (BESA) Research v7.0. The MRI artifacts were removed using the BESA fMRI artifact removal module’s implementation of the Allen method (Allen et al., 2000). The parameters used for artifact removal were 16 artifact averages on fMRI data collected with a TR of 2,000 ms. The correction was done using either the MR pulse trigger or phase synchronization between the EEG and MRI acquisition hardware.

Electroencephalogram data were visually inspected, and muscle artifacts were removed by trained research assistants. Exceptionally noisy electrode channels were interpolated. For ERP generation, a low cutoff filter of 0.1 Hz was applied, and the ERP baseline was defined using the 100 ms preceding stimulus onset. After generating the ERPs, the blink and BCG artifacts were removed and a high cutoff filter of 40 Hz was applied. Eye-blink and BCG artifacts were removed using defined topographies. A data block containing a stereotypical artifact was marked and either defined as an eye-blink or BCG. Then a pattern matching algorithm in BESA selected the independent components analysis channel that matched for the highest explained variance (∼95%), and subsequently used to remove the artifact (Berg and Scherg, 1994). For eye-blink correction, the data were filtered between 1 and 12 Hz. For BCG correction, the data were filtered between 1 and 20 Hz, and a zero-phase filter slope was used. For low cutoff, the filter type was set at 12 dB/oct and for high cutoff the filter type was set at 24 dB/oct.

After artifact cleaning, the mean number of stimuli across all trials that contributed to the ERP averages was 277 (SD = 30) and 282 (SD = 13) for the low- and high-load WM delay period conditions, respectively (paired *t*-test *p* = 0.354, n.s.). The mean number of stimuli across all trials that contributed to the ERP averages was 92 (SD = 8.3) and 236 (SD = 15.5) for the low- and high-load WM encoding period conditions, respectively (*p* < 0.001). This significant difference for encoding exists because, by definition, high- vs. low-load encoding involves presentation of more stimuli (five vs. two scenes). EEG signals were segmented in epochs around stimulus onset for 1,000 ms at the start of the encoding period for the encoding period analysis and at the start of the delay period for the delay period analysis. Then the artifact-corrected epochs were averaged for each condition (low- and high-load) and task period (encoding and delay). The average ERPs for each condition and task period were then used as input for a group ERP statistical analysis performed with BESA Statistics v2.0 with appropriate multiple comparisons corrections across space and time using random permutation testing (Maris, 2012).

### 2.4. MRI acquisition and analysis

High-resolution structural MRI volumes were acquired for each participant including a T1-weighted volume (TE 2.12/TR 2,400, 254 mm FOV, 1 mm^3^ voxels), a T2-weighted volume (TE 408/TR 2,200, 254 mm FOV, 1 mm^3^ voxels), and PETRA volume (TE 0.07/TR 3.61, 298 mm FOV, 0.938 mm^3^ voxels). The T1 and T2 volumes were used to build custom realistic head models (scalp, skull, CSF, and brain) for each participant, while the PETRA volume was used to visualize and localize electrode locations on the scalp. The functional MRI data collected simultaneously with the EEG acquisition included blood oxygen level dependent echo planar images (BOLD-EPI, TE 30/TR 2,000, FOV 249 mm, 35 axial slices, 3 mm^3^ voxels).

Magnetic resonance imaging data were analyzed using Analysis of Functional Neuro Images (AFNI) software (Cox, 1996). Each BOLD-EPI four dimensional timeseries was aligned to the T1-weighted structural volume using AFNI’s align_epi_anat.py script, which was also used to perform automated skull-stripping of the T1 volume, EPI slice timing correction, alignment of the EPI to T1 volume using a 12 parameter affine transformation and spatial blurring of the EPI timeseries using a Gaussian full width at half maximum of 4 mm. First (subject) level statistical analysis of the processed individual subject EPI timeseries was performed using AFNI’s 3dDeconvolve with WM task phase of 7 s for high load encoding, 2 s for low load encoding, 6 s for the delay period, and 1.4 s for the probe period. These regressors were convolved with a hemodyamic response function (HRF) of the form *HRF(t*) = *int(g(t-s), s* = *0..min(t,d)) where g(t*) = *t^∧^q × exp(-t)/(q^∧^q × exp(-q)) and q* = 4 and then added to a general linear model design matrix that included them as regressors of interest. Regressors of no interest in the design matrix included polynomial functions to model baseline shifts with a cutoff of (p-2)/D Hz where D is the duration of the imaging run and the three translation and three rotational subject motion parameters. Second (group) level statistical maps were computed using AFNI’s 3dMEMA using each subject’s voxelwise regression coefficient maps to test first for the main effect of task phase (encoding, delay, and probe) and second to compare encoding (scenes) vs. delay (scrambled scenes) as a function of WM load.

### 2.5. fMRI-weighted source analysis

The first level comparisons of encoding (scenes) vs. delay (scrambled scenes) between high and for low WM load were output as individual subject maps in Talairach space with 2 mm isotropic resolution and thresholded using a false discovery rate of *q* = 0.01. They were then imported into BESA Research 7.0 as functional activation weight maps for constrained dipole source analysis (Scherg, 1990).

For each participant, the scalp positions of the electrodes used in the simultaneous EEG-fMRI scanning sessions were estimated initially using an approximation of locations from a standard montage template (BESA-MRI-Standard-Electrodes) and then adjusted manually based on visual inspection of the indentation-artifacts caused by electrode on the scalp, which appeared as dips on the scalp surface reconstructions. An example of electrode locations for a single subject is shown in Supplementary Figure 2. Each participant’s T1-weighted anatomical MRI was segmented manually in BESA MRI v2.0 to create a 4-layer Finite Element Model (FEM) realistic head model to be used in the source analysis. Based on individual electrode coordinates, segmentation with anatomical landmarks transformed to Talairach Space, and fMRI statistical maps imported for each condition, BESA calculated the best fitting ellipsoid of each participant (Scherg, 1992). The fMRI-informed regional EEG source estimation with anatomical constraints approach has been documented to be a better modeling than seeding dipoles based solely on anatomical locations (Phillips et al., 2002; Ahlfors and Simpson, 2004; Ou et al., 2010).

Seed-based dipole fitting was based on *a priori* hypotheses to explain ERP changes as a function of task period and WM load. For encoding, two equivalent dipoles were fitted onto bilateral parahippocampal cortex (PHC) for each participant at low and high load WM conditions. For delay, two equivalent dipoles were fitted onto bilateral thalamus. For each participant, a time window was chosen from onset to the peak of the first Global Field Potential (GFP) peak, which is a measure for spatial standard deviation as a function of time (Strik and Lehmann, 1993). An example of a single participant’s GFP waveform is shown in Supplementary Figure 3 and an example analysis window used in the source analysis is shown in Supplementary Figure 4. During seeding of dipole locations, weighting with fMRI activation maps was initially turned off to avoid potential bias in determining the initial seed location. The dipoles were then fit onto the respective sources weighted by the fMRI statistical map activation using the RAP-MUSIC algorithm as implemented in BESA source space that estimates the dipole locations using the weighted fMRI images (Grech et al., 2008). The dipole positions were constrained to stay within the target regions, but their orientations were kept free before the fit. All the dipoles fell within the appropriate brain regions (PHC and thalamus) after the fit. An example fit with fMRI weighting for thalamus is shown in Supplementary Figure 5. The dipole positions were expressed as Talairach coordinates in units of millimeters (mm) and averaged across all subjects. The source waveforms for each participant and condition were exported and then imported for group source statistical analyses in BESA Statistics v2.0.

## 3. Results

### 3.1. Behavioral

Percent correct accuracy was significantly better for the low- compared to high-load WM task (90.64%, SD 19.44 vs. 78.27, SD 25.96, related-samples Wilcoxon Signed Rank test *p* = 0.002). Reaction times were significantly faster for the low- compared to high-load WM task (855.40 ms, SD = 197.13 vs. 882.50, SD = 279.82, related-samples Wilcoxon Signed Rank test *p* = 0.033). In the pilot behavioral experiment, performance was better for both loads when the delay period consisted of a fixation cross compared to scrambled stimuli (Supplementary Figure 6).

### 3.2. fMRI

**FIGURE 2:**
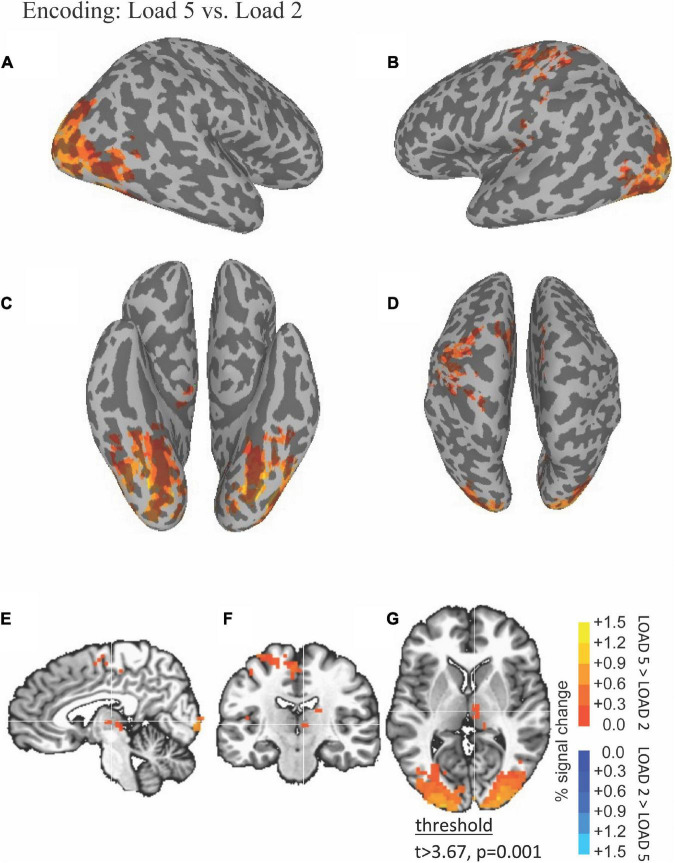
Functional magnetic resonance imaging (fMRI) Differences Between High and Low Load during the Encoding Period. Thresholded fMRI statistical maps (*t* > 3.67, *p* = 0.001, and cluster size > 40 voxels) displayed on inflated cortical surface representations [**(A)** left hemisphere, **(B)** right hemisphere, **(C)** ventral view, and **(D)** dorsal view] and orthogonal views [**(E)** sagittal, **(F)** coronal, and **(G)** axial view]. The crosshairs in the orthogonal view is located *x* = 28, *y* = 38, and *z* = 48 mm, the peak location of a cluster of activity in medial dorsal nucleus of the thalamus that was greater for high compared to low load encoding.

The ability of fMRI to discriminate among the WM encoding is illustrated by results shown in Figure 2. Right thalamus was more active during high (five scenes) compared to low (two scenes) load encoding (red in Figures 2E–G).

**FIGURE 3:**
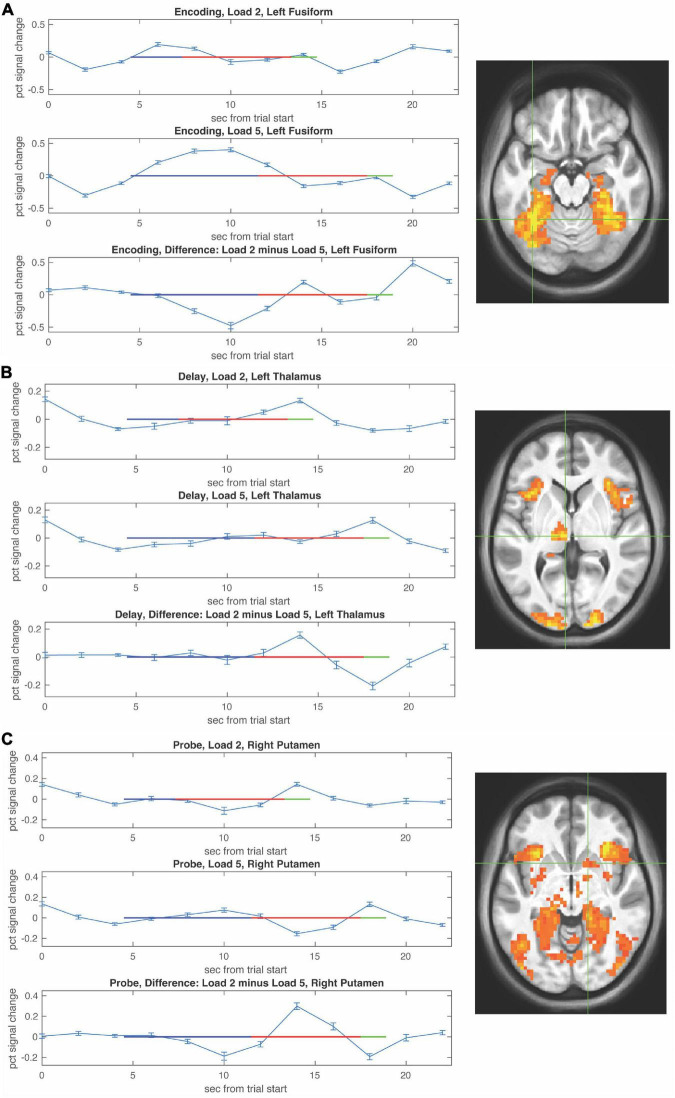
Functional magnetic resonance imaging (fMRI) timecourses as a function of load during the encoding, delay, and probe periods. Timecourses (mean ± S.E.M) are shown for local maxima during encoding in left fusiform **(A)**, during the delay period in left thalamus **(B)**, and during the probe choice period in right putamen **(C)**. For each timecourse low load is shown in the upper panel, high load is shown in the middle panel, and the difference (low–high) is shown in the bottom panel. The encoding period is depicted by a blue horizontal line, the delay period by a red horizontal line, and the probe period by a green horizontal line with beginning of the lines shifted by the hemodynamic response.

Functional magnetic resonance imaging-BOLD activity increases during WM encoding in fusiform with greater activity at higher WM load (Figure 3A).

Table 1 lists the cluster sizes, coordinates, and brain regions with significant differences between high and low load during the encoding period.

**TABLE 1:** Brain regions with significant differences between high and low load during the encoding period.

Cluster	Cluster size	*X*	*Y*	*Z*	Brain region
1 (L5)	1,040	29	−68	−12	R Lingual gyrus
2 (L5)	921	−31	−89	−6	L Middle occipital gyrus
3 (L5)	297	−31	−14	63	L Precentral gyrus
4 (L5)	45	28	38	48	R Thalamus
5 (L5)	45	−46	−23	18	L Supramarginal gyrus

Cluster sizes after thresholding at *t* > 3.67 (*p* < 0.001) are reported as number of contiguous voxels in descending order. In parentheses after the cluster number it is indicated whether the activity in the cluster was greater at encoding during the high (L5) or low (L2) load condition. Here all five clusters showed greater activity during high load encoding compared with low load encoding. For each cluster, the *x*, *y*, *z* Talairach coordinate is reported in mm for the peak local maxima within the cluster followed by the labeled brain region.

**FIGURE 4:**
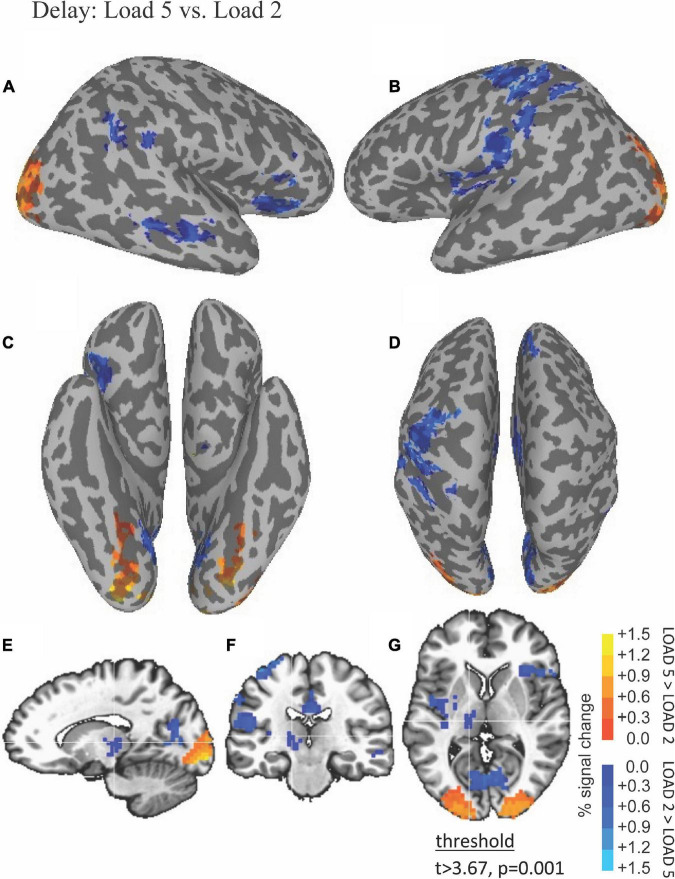
Functional magnetic resonance imaging (fMRI) differences between high and low load during the delay period. Thresholded fMRI statistical maps (*t* > 3.67, *p* = 0.001, and cluster size > 40 voxels) displayed on inflated cortical surface representations [**(A)** left hemisphere, **(B)** right hemisphere, **(C)** ventral view, and **(D)** dorsal view] and orthogonal views [**(E)** sagittal, **(F)** coronal, and **(G)** axial view]. The crosshairs in the orthogonal view is located *x* = –10, *y* = –17, and *z* = 12 mm, the peak location of a cluster of activity in medial dorsal nucleus of the thalamus that was greater for low compared to high load during the delay period.

The ability of fMRI to discriminate among the WM maintenance is illustrated by results shown in Figure 4. Left thalamus was more active during low compared to high load during the delay period (blue in Figures 4E–G).

Activity during the WM delay period ramped up in thalamus more so at low WM load (Figure 3B, top) compared to high WM load (Figure 3B, bottom). Activity during the probe choice increased in putamen reaching a similar peak for both low WM load (Figure 3C, top) and high WM load (Figure 3C, bottom).

Table 2 lists the cluster sizes, coordinates, and brain regions with significant differences between high and low load during the delay period.

**TABLE 2:** Brain regions with significant differences between high and low load during the delay period.

Cluster	Cluster size	*X*	*Y*	*Z*	Brain region
1 (L5)	472	−10	−92	−6	L Calcarine gyrus
2 (L2)	447	2	−68	9	R Cuneus
3 (L5)	431	29	−86	−9	R Inferior occipital gyrus
4 (L2)	421	−37	−26	63	L Precentral gyrus
5 (L2)	175	−46	−23	18	L Supramarginal gyrus
6 (L2)	127	44	19	−3	R Inf front gyrus (p. Orbitalis)
7 (L2)	120	5	31	57	R Superior medial gyrus
8 (L2)	75	65	−32	−3	R Middle temporal gyrus
9 (L2)	67	2	−23	27	R Middle cingulate cortex
10 (L2)	59	56	−53	33	R Angular gyrus
11 (L2)	44	−10	−17	12	L Thalamus

Cluster sizes after thresholding at *t* > 3.67 (*p* < 0.001) are reported as number of contiguous voxels in descending order. In parentheses after the cluster number it is indicated whether the activity in the cluster was greater in the delay period during the high (L5) or low (L2) load condition. In this comparison two clusters showed greater activity in high load delay period compared with the low load delay period, while nine clusters showed greater activity in the low load delay period compared with the high load delay period. For each cluster, the *x*, *y*, *z* Talairach coordinate in mm is reported for the peak local maxima within the cluster followed by the labeled brain region.

### 3.3. fMRI-weighted EEG source analysis

For delay conditions, the average Talairach coordinates for left hemisphere fitted dipoles were *x* = −13.4 mm, *y* = −21.9 mm, *z* = 3.7 mm, and right hemisphere fitted dipoles were *x* = 11.6, *y* = −21.8, and *z* = 3.8. Left and right hemisphere dipole coordinates during delay period always fell within the thalamus for all participants. For the encoding period, the average Talairach coordinates for the left hemisphere were *x* = −25.3, *y* = −38.1, *z* = −9.6, and for the right hemisphere were *x* = 24.7, *y* = −37.9, and *z* = −9.4. Left and right dipole coordinates during the encoding period always fell within the PHC for all participants. Tables 3, 4 list the individual coordinates for all the participants included in the source analysis during the delay and encoding periods, respectively.

**TABLE 3:** Individual Talairach coordinates (mm) of functional magnetic resonance imaging (fMRI)-weighted dipole fits for the delay condition.

	Right			Left		
	***x***	***y***	***z***	***x***	***y***	***z***
Participant 1	13.4	-25.3	0.5	-18.8	-25.6	1.6
Participant 3	13.4	-25.3	0.5	-17.8	-25.2	1.2
Participant 5	13.4	-25.3	0.5	-15.6	-27.4	0.5
Participant 6	13.4	-25.3	0.5	-15.6	-25.6	4
Participant 7	13.4	-25.3	0.5	-18.8	-25.3	-0.5
Participant 8	−6	−9	6	−6	−9	6
Participant 9	9.1	-14.5	1.6	-15.6	-14.5	0.5
Participant 10	17.8	-26.8	8.5	-10.2	-27.4	4.8
Participant 11	8.1	-15.3	4.8	-11.3	-15.3	2.7
Participant 12	15.1	-21.7	2.8	-15.6	-21.7	5.9
Participant 13	14.5	-26.4	5.9	-13.4	-26.4	4.8
Participant 14	16.1	-30.7	2.6	-14.5	-30.7	3.8
Participant 15	12.4	-21.2	4.8	-14.3	-21.2	3.8
Participant 16	6	−9	6	−6	−9	6
Participant 17	12.5	-29.5	2.9	-15.3	-29.8	4
Participant 18	6	−9	6	−6	−9	6
Participant 19	12.4	−21	4.8	-13.4	−21	4.8
Participant 20	19.2	-33.1	3.2	-14.1	-33.2	4.3
Participant 21	14.5	-27.4	4.8	-18.8	-27.4	3.8
Participant 22	11.3	-19.9	0.5	-10.2	-19.9	1.7
Participant 23	9.1	-17.8	4.8	-9.1	-17.8	5.9
Participant 24	10.2	-19.9	4.8	-13.4	-19.9	4.8
Average:	11.6	-21.8	3.5	-13.4	-21.9	3.7

**TABLE 4:** Individual Talairach coordinates (mm) of functional magnetic resonance imaging (fMRI)-weighted dipole fits for the encoding condition.

	Right			Left		
	***x***	***y***	**z**	***x***	***y***	***z***
Participant 1	25.3	-35	-10.2	-25.3	-35	-9.1
Participant 3	26.4	-42.8	-6.5	-24.2	-58.6	-5.9
Participant 5	23.1	-58.6	-5.9	-27.4	-58.6	-5.9
Participant 6	28.4	-26.4	-14	-35	-26.4	-14.5
Participant 7	26.4	-47.3	-5.9	-22.1	-47.3	-5.9
Participant 8	25.6	-58.6	-5.6	−21	-31.7	-8.1
Participant 9	21	-31.7	-11.3	-26.4	-31.7	-10.2
Participant 10	22.1	-28.5	-17.8	-24.2	-28.5	-18.8
Participant 11	25.3	-42.5	-8.1	-32.8	-42.5	-8.1
Participant 12	25.3	−50	-4.8	-23.1	−50	-4.8
Participant 13	23.1	−35	-8.1	-26.4	−35	-10.2
Participant 14	24.2	-17.8	-19.9	-27.4	-17.8	-22.1
Participant 15	25.3	−50	-5.9	−21	-50	−7
Participant 16	21.2	-32.8	-9.8	-21.2	-32.8	-9.8
Participant 17	23.1	-31.7	-9.1	-22.1	-31.7	-9.1
Participant 18	24	-29	-12.6	−24	-45.4	-10.1
Participant 19	24.2	-43.6	-7.2	-25.3	-42.5	-7.2
Participant 20	24.2	-28.5	-12.4	-25.3	-28.5	-12.4
Participant 21	26.4	-41.4	-8.1	-24.2	-40.3	-8.1
Participant 22	26.8	-35.1	-6.3	-23.3	-34.8	-7.2
Participant 23	28.5	-37.1	−7	-25.3	-38.2	−7
Participant 24	23.1	-30.7	-10.2	-29.6	-31.5	-9.7
Average:	24.7	-37.9	-9.4	-25.3	-38.1	-9.6

**FIGURE 5:**
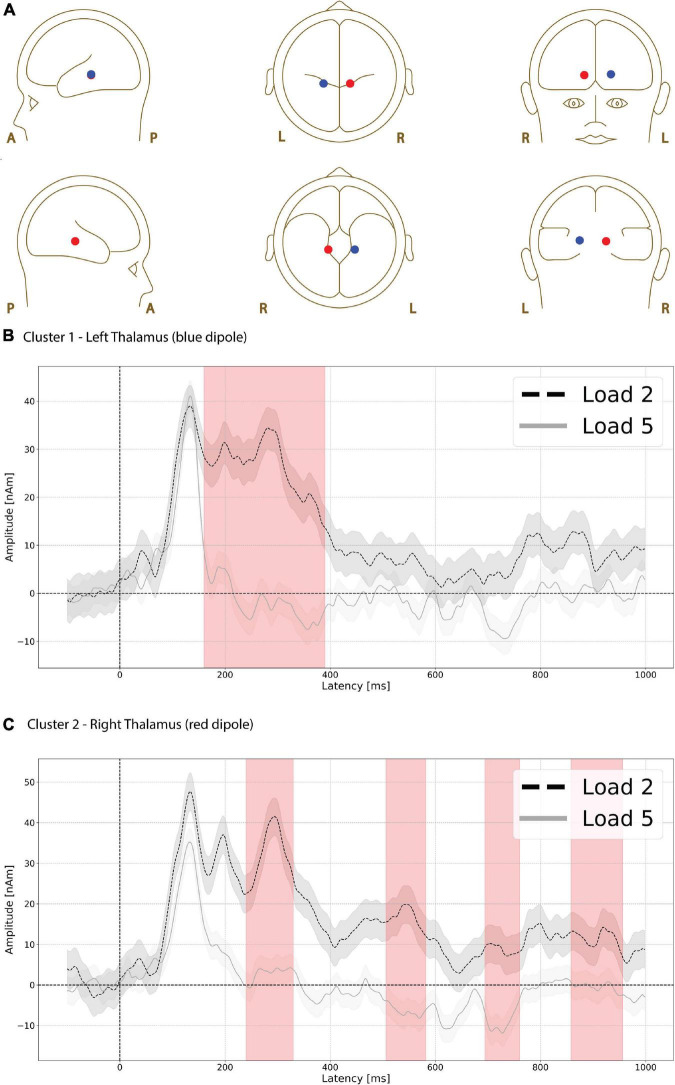
Functional magnetic resonance imaging (fMRI)-weighted thalamic source group EEG waveforms during the delay period. Head plot of asymmetric dipole clusters located in thalamus in left (blue) and right (red) hemispheres **(A)**. Cluster 1 in left thalamus (blue dipole) showed evoked responses that were significantly higher (*p* = 0.003) in amplitude during the low compared to high load delay period (red shading) between 160 and 390 ms **(B)**. Cluster 2 in right thalamus also showed greater amplitude during low compared to high load in four separate time windows **(C)**: the earliest window of significant difference was between 240 and 330 ms (*p* = 0.022, first red vertical shade), followed by an interval between 506 and 582 ms (*p* = 0.034 second red vertical shade), followed by 694–760 ms (*p* = 0.046, third red vertical shade), and finally 858–956 ms (*p* = 0.021, fourth red vertical shade). Lines are mean amplitude with shaded error bars representing ±95% confidence intervals.

Functional magnetic resonance imaging-weighted sources in left and right thalamus (Figure 5A, left is red, right is blue) showed evoked responses that were significantly higher in amplitude during the low compared to high load delay period (Figure 5B, red shading). The difference was greatest in left thalamus (*p* = 0.003, max *t*-value 3.052, latency at max 170 ms, load 2 mean 26.248 nAm, load 5 mean −1.746 nAm, Figure 5B) between 160 and 390 ms.

The right thalamus source also showed greater amplitude during low compared to high load in four separate time windows. The earliest window of significant difference was between 240 and 330 ms (*p* = 0.022, max *t*-value 3.679, latency at max 290 ms, load 2 mean 32.518 nAm, load 5 mean 2.919 nAm Figure 5C, first red vertical shade) followed by time intervals between 506 and 582 ms (*p* = 0.034, max *t*-value 3.314, latency at max 538 ms, load 2 mean 16.831 nAm, load 5 mean −6.520 nAm, Figure 5C, 2nd red vertical shade), 694 and 760 ms (*p* = 0.046, max *t*-value 2.808, latency at max 726 ms, load 2 mean 8.727 nAm, load 5 mean −9.242 nAm, Figure 5C, 3rd red vertical shade), and 858–956 ms (*p* = 0.021, max *t*-value 3.491, latency at max 914 ms, load 2 mean 11.564 nAm, load 5 mean −0.561 nAm Figure 5C, 4th red vertical shade).

Functional magnetic resonance imaging-weighted sources in left and right parahippocampus (Supplementary Figure 7a) showed similar evoked response amplitude during the low and high load encoding periods with no significant differences between load (*p* = 0.486, max *t*-value 2.357, latency at max 86 ms, load 2 mean 31.071 nAm, load 5 mean 18.205 nAm) at any time period in left or right hemisphere (Supplementary Figures 7b,c).

**FIGURE 6:**
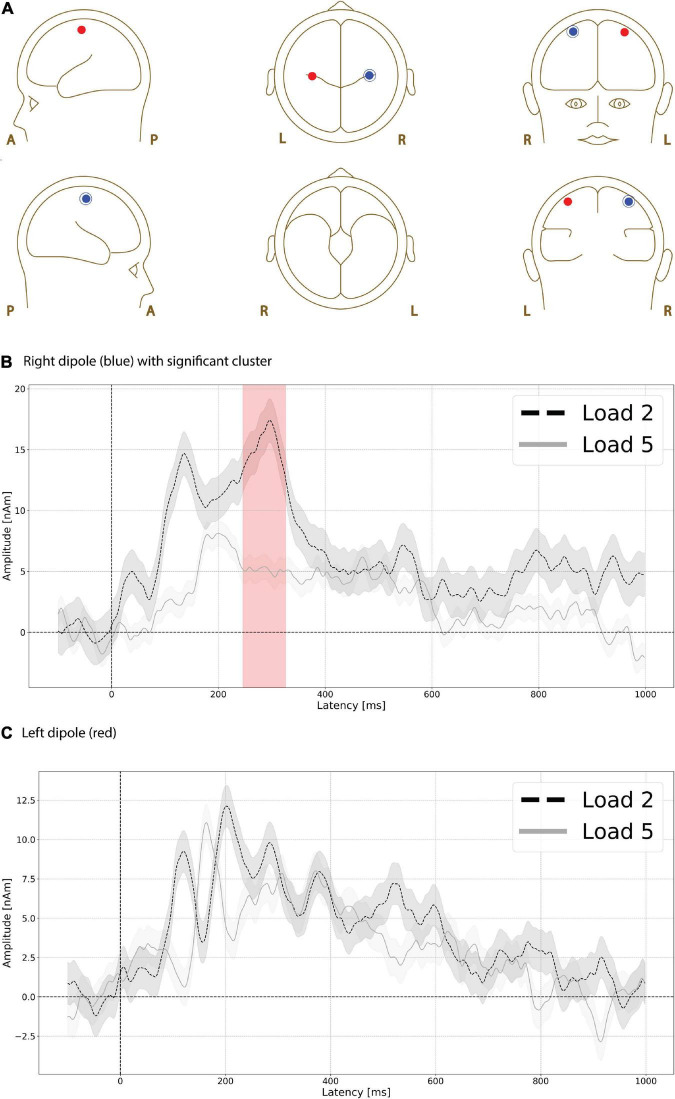
Functional magnetic resonance imaging (fMRI)-weighted motor cortex source group EEG waveforms during the delay period. Head plot of asymmetric dipole clusters located in left motor cortex (red) and right (left) hemispheres **(A)**. Evoked responses were significantly higher (*p* < 0.01) in amplitude during the low compared to high load delay period in right motor cortex at ∼250 ms (**B**, red shading), but no significant differences were found at any time interval in left motor cortex **(C)**. Lines are mean amplitude with shaded error bars representing ± 95% confidence intervals.

Functional magnetic resonance imaging-weighted sources in left and right motor cortex (Figure 6A, left is red, right is blue) showed evoked responses that were higher in amplitude during the low compared to high load delay period. A significant difference (*p* = 0.035, max *t*-value 3.243, latency at max 300 ms, load 2 mean 15.312 nAm, load 5 mean 5.081 nAm) was found between low and high load delay period in right motor cortex (Figure 6B) at ∼250 ms (Figure 6B, red shading), but no significant differences were found at any time interval in left motor cortex (Figure 6C).

### 3.4. Source EEG-behavior correlations

Since there were significant differences in the bilateral thalamus as a function of WM load, we tested whether source EEG correlated with the behavioral performance. The individual subject grand source waveforms for low- and high-load delay conditions were correlated with the percent correct task accuracy. No statistically significant clusters that correlated with performance were found at either low (*p* = 0.475) or high load (*p* = 0.256). Individual subject grand source waveforms from both the delay conditions were also correlated with reaction times, but no statistically significant clusters (*p* > 0.05) were found. To further explore the relationship between source activity and performance, classification was performed using linear discriminant analysis with thalamus source amplitudes during the delay period and task performance as predictors and the low or high load condition as class labels. Classification accuracy was 73.68% with 26.32% of datapoints misclassified by the linear discriminant function (Supplementary Figures 8–10).

## 4. Discussion

Previous studies in humans have focused on measuring the delay activity associated with a limited capacity WM buffer in prefrontal and posterior brain regions and have found that maintaining more items (i.e., higher load) leads to increases in activity (Rypma et al., 1999; Schon et al., 2009). Relatively less is understood about the role of the thalamus, a subcortical structure connected to prefrontal cortex that is documented also to play a critical role in WM (Guo et al., 2017; Inagaki et al., 2019). In the present study we used a scene WM task and examined delay activity using fMRI and source estimated EEG during low and high load maintenance. During the delay period, participants viewed scrambled scenes which served as both a perceptual baseline and as interfering stimuli. The main result found was differential activation in bilateral thalamus such that greater activity measured by both fMRI and EEG was found during low load, while less activity was found during high load maintenance. If the role of thalamus, specifically Mdt, is to facilitate WM, then as load increased so should delay activity. Instead, we found the opposite suggesting alternatively that the ability of thalamus to suppress sensory input in the form of the scrambled scenes during WM maintenance is load dependent with a reduced thalamus response reflecting an inability to filter interfering stimuli when there are high demands on maintenance. This reduced inability to filter was confirmed by worse performance on the high load task despite the number of interfering stimuli presented during the delay being equal between the high and low load conditions.

A sensory gating mechanism could explain differential activity in the thalamus as a function of load. The thalamus could regulate the gain of sensory processing during the different loads such that the processing of sensory information (e.g., scrambled scenes) is down regulated when this information interferes with the maintained information due to capacity limits reached during the high load condition. On the other hand, sensory processing of the scrambled scenes may be upregulated when this information might not interfere with maintained scenes during the low load condition, when there are fewer demands on capacity. Detection of interfering stimuli could trigger activity in dlPFC via thalamus relays (Knight et al., 1999). The PFC has also been conceptualized as a dynamic filtering mechanism (Shimamura, 2000). The thalamus could work with PFC to apply a dynamic filter to select information based on current task requirements, with reciprocal connections between thalamus and PFC supporting such a role. Evidence suggest that dlPFC is involved in goal-based control by inhibition of task-irrelevant information (Ridderinkhof et al., 2004). Using transcranial magnetic stimulation (TMS), goal-based representations in PFC were used to modulate how perceptual information is selectively filtered such that the task goal specified by an instruction could modulate perceptual processing by inhibiting task-relevant information (Feredoes et al., 2011). Thalamic projects to PFC could inhibit task-irrelevant information in service of cognitive control.

In demanding WM tasks like the N-back, the dlPFC network expands showing marked connectivity with parietal regions and areas of the ventral visual pathway (Cohen and D’Esposito, 2016). When WM load increases, the thalamus may signal to the PFC to increase the connection strength of item representations to become greater across networks, including parahippocampal, parietal, and visual areas. We find support for this as our fMRI results show increases in cortical regions during high load maintenance despite relatively less thalamic activity at high compared to low load. Based on the present results, we hypothesize that the thalamus may be enhancing the task-relevant information or inhibiting task-irrelevant information. On this account, during the high-load condition, the thalamus may serve to inhibit distracting information to activate relevant stimuli information in higher cortical areas like the primary visual cortex. This account would help explain our results from the study combining both fMRI and EEG methods. The thalamus may be involved in successfully orchestrating inhibitory control when high-load information is being maintained. Therefore, the thalamic responses could be attenuated to suppress the complex environmental stimuli. The differential thalamic activation could also explain the difference in behavioral performance in both loads. The highest evoked activity during maintenance of fewer stimuli in the presence of interfering perceptual stimuli implies better consolidation of stimuli, which eventually leads to better performance (Lavie et al., 2004). Alternatively, the thalamus, with its reciprocal connections with PFC and motor areas, may serve to prepare the participant for the appropriate behavioral response (Fonken et al., 2016). On this account, higher thalamic activation could mean more increased preparedness and readiness to make a behavioral response during the low-load condition. This account would also explain that lower thalamic activation would imply reduced confidence and readiness to make the relevant behavioral response because more scenes had to be maintained during the higher load condition.

Future studies should seek replication in modalities other than visual and seek to examine how differential thalamic activation and connectivity relates to behavioral performance under a range of different WM loads to equate performance. Further research will also be necessary to determine the degree to which WM maintenance mechanisms reflect the selection of task-relevant information vs. the inhibition of irrelevant information. This idea could be tested using category-specific stimuli, where the participants would be asked to remember or ignore specific categories during each trial. Furthermore, it would also be interesting to test for differences in thalamic activations as a function of delay period length. By increasing the delay period duration, one could find evidence whether the thalamic activation rises just before the behavioral response when the end of the delay period is unpredictable. Future studies should further test whether there is truly a negative correlation between thalamus during the delay activity and posterior cortical areas during encoding and its relation to successful retrieval of items.

In summary, the present study results suggest that thalamus is involved in working memory and is differentially active as a function of visual WM load. When the WM load is low, the thalamus is more active than when WM load is high. During high load, the thalamus may function to attenuate incoming distracting perceptual stimuli while during low load the thalamus shows less attenuation in the face of distracting perceptual input. This suggests that the thalamus is preparing the superficial cortical areas for successful task-relevant information during high load WM maintenance. More research is needed to understand how the thalamus and PFC work in concert to inhibit potentially disruptive or irrelevant information and modulate attention during maintenance in the working memory delay period.

## Data availability statement

The raw data supporting the conclusions of this article will be made available by the authors, without undue reservation.

## Ethics statement

The studies involving human participants were reviewed and approved by The City University of New York Human Research Protection Program (CUNY HRPP IRB). The patients/participants provided their written informed consent to participate in this study.

## Author contributions

TE: conceptualization, funding acquisition, project administration, resources, supervision, visualization, formal analysis, writing—original draft, and writing—review and editing. BG, JO, and CP: data curation, investigation, formal analysis, visualization, writing—original draft, and writing—review and editing. All authors contributed to the article and approved the submitted version.
