# Supplementary material for: A simultaneous EEG-fMRI study of thalamic load-dependent working memory delay period activity
Source: Front Behav Neurosci. 2023 Feb 23;17:1132061. doi: 10.3389/fnbeh.2023.1132061 (PMC9997713; doi:10.3389/fnbeh.2023.1132061)
Supplement: Supplementary file 1 [file Data_Sheet_1.PDF]

Supplementary Material

Materials and Methods

Pilot Behavioral Experiment

The experimental design of the main EEG study (described in *Methods*) was motivated by the results of a separate pilot behavioral study in which a different group of participants completed a Sternberg working memory (WM) task with a similar design. The paradigm consisted of two conditions, a low-load condition (two stimuli, *Supplementary Figure 1a*) and a high-load WM condition (five stimuli, *Supplementary Figure 1b*).

Participants completed trials with both loads in randomized order, followed by an immediate recognition task and a separate long-term recognition task 24 hours later. During the WM trials, participants saw either a standard fixation cross or phase-scrambled scenes during the delay period.

A) Low Load Working Memory Condition with Delay Period Scrambled Stimuli as Interference

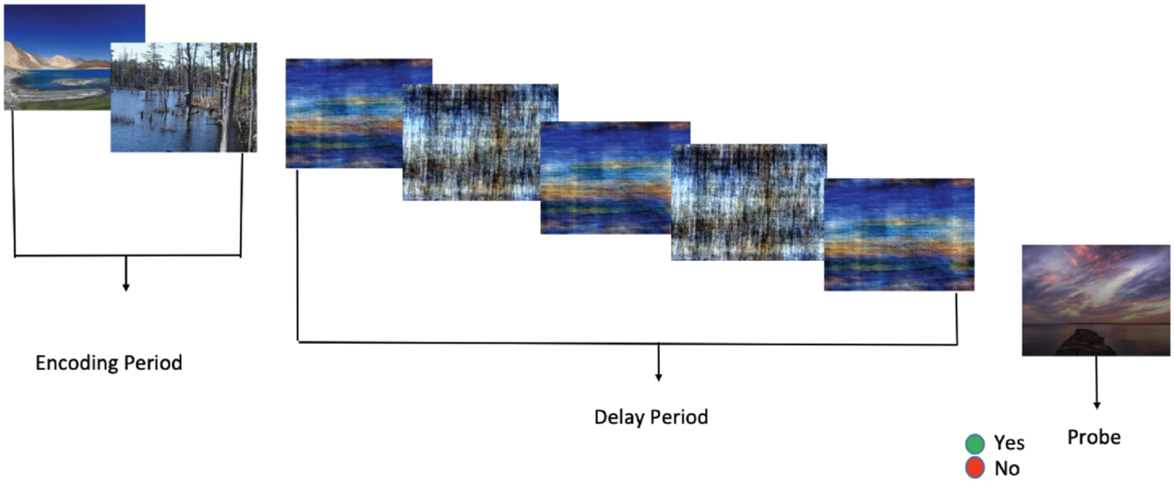

B) High Load Working Memory Condition with Delay Period Scrambled Stimuli as Interference

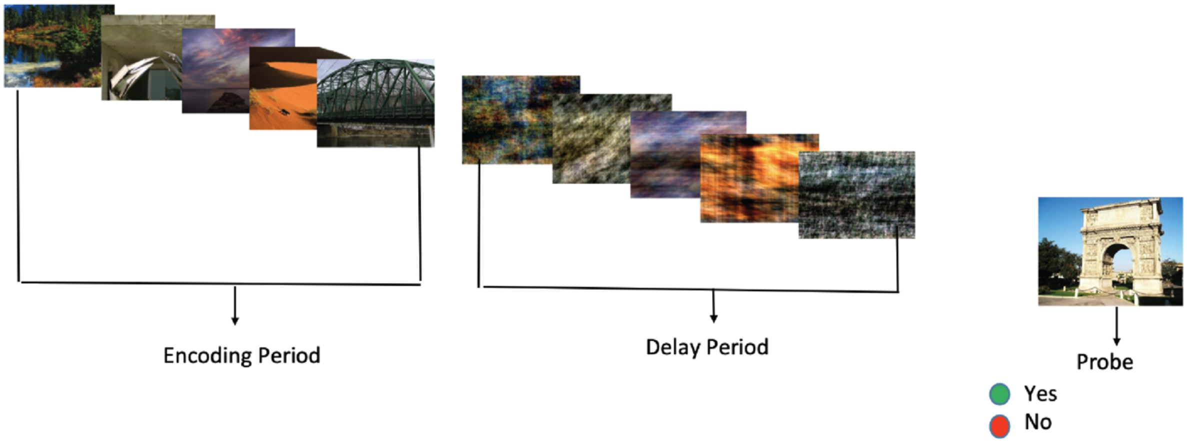

Supplementary Figure 1. Pilot Behavioral Experiment Paradigm. An example trial for the low load WM condition with scrambled stimuli during presented during the delay period (a). Two scene stimuli were presented during the encoding period, followed by phase scrambled versions of the scenes, and then a probe stimulus to which the subject signaled whether they did or did not remember it from the set of scenes presented during encoding. An example trial of the high load WM condition with scrambled stimuli during the delay (b). In the high load condition, five images were presented during the encoding period, followed by phase scrambled versions of the scenes, and then a probe to which the subject answered whether they remembered it from the set of scenes presented at encoding. In a separate condition, participants saw a standard fixation cross (not shown) instead of scrambled scenes, which allowed for subsequent comparison of task performance as a function of the type of filler during the delay period.

fMRI-Weighted Source Analysis

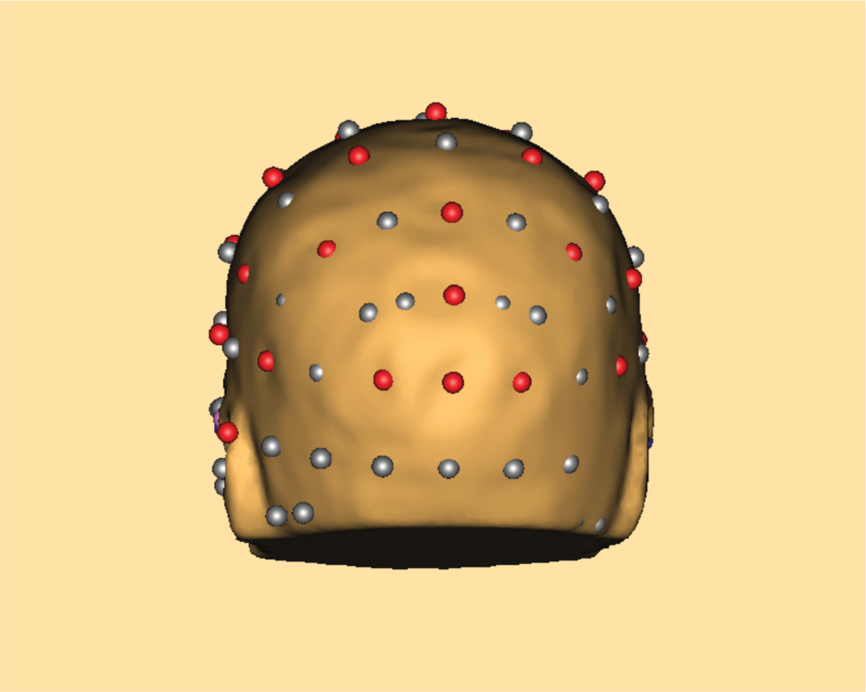

Supplementary Figure 2. Example of electrode-localization superimposed on a single-subject scalp surface reconstruction. Standard set of 3D electrode positions based on the 10-10 electrode system was used to coregister electrodes with individual scalp surface reconstructions. Electrode positions were visually inspected to make sure the electrodes were as close to the indentation artifacts on the scalp caused by the electrode gel. An example from a single subject is shown above.

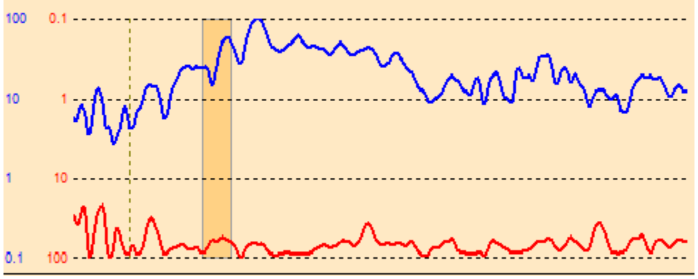

Supplementary Figure 3. Example Global Field Power for Source Analysis. GFP (Global Field Power) of the original waveform of a single subject representing all combined delay activity of the experiment (blue) is displayed in logarithmic scale. The unexplained fraction of the data variance, or Residual Variance (RV) is also displayed (red) in inverted logarithmic scale. The fit process finds a source model that minimizes this RV. RV = 62% and length of duration of the x-axis is 1000 msecs.

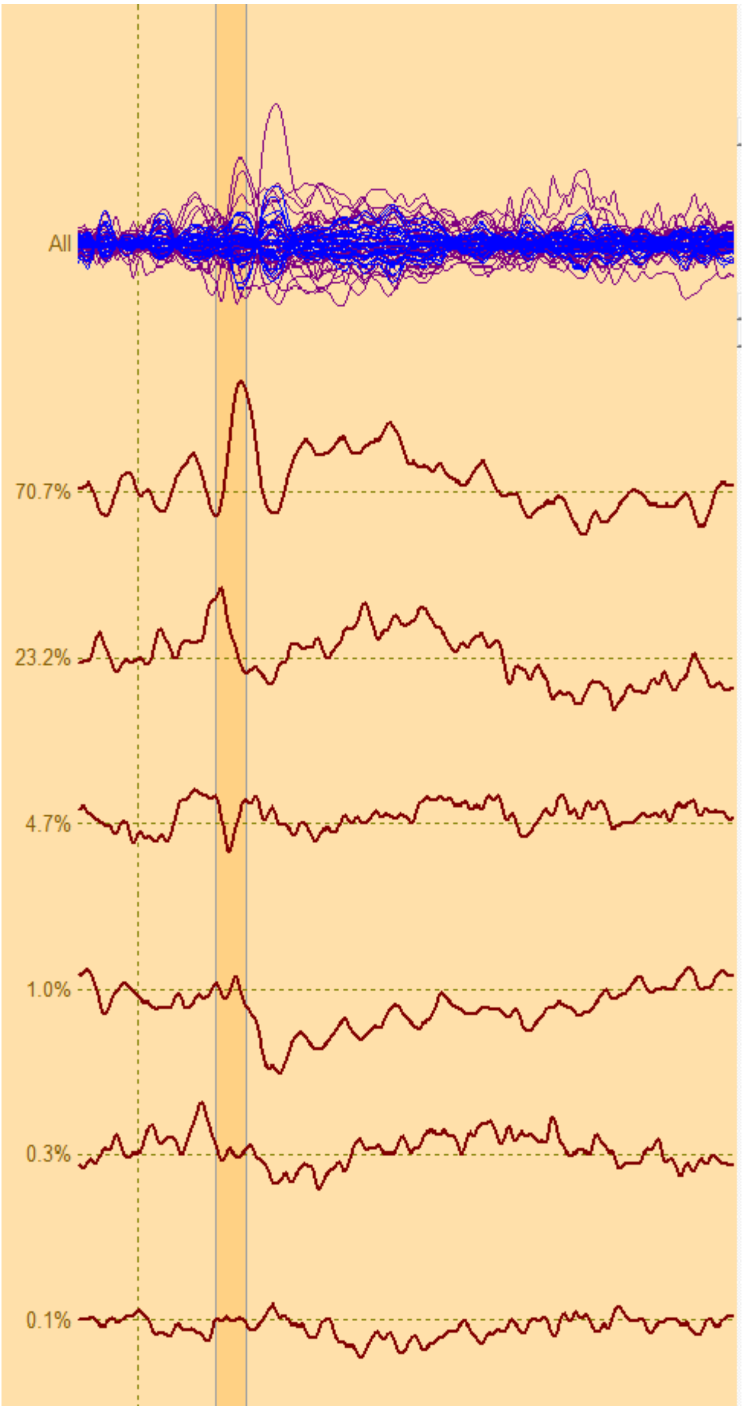

Supplementary Figure 4. Example analysis window used in the source analysis. The upper trace shows the butterfly-plot of the averaged spike-signal (epoch duration: -100 msec to 998 msec; the dotted line represents the stimulus onset). The model waveforms generated by the current source model is shown in blue. The following traces show the source-waveforms corresponding to the detected component (highlighted); numbers on the left indicate the contributed variance of each component to the measured signal. Note that one component accounts for more than 70% of the signal on the ascending slope.

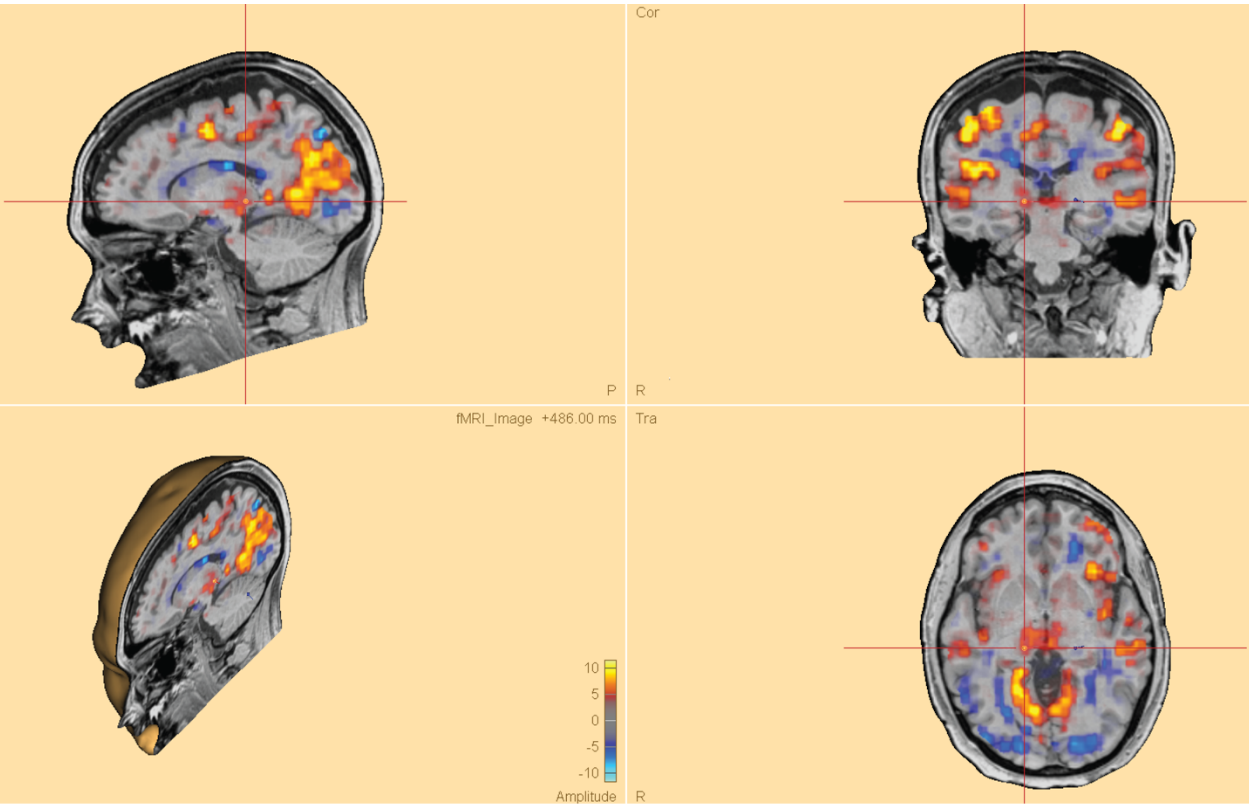

Supplementary Figure 5. Dipole Position Example. Source Localization (*a priori* dipole modeling) highlighting the right (red; Talairach coordinates: x = -18.8, y = -25.3, z = 0.5) thalamus. Note that the realistic head model was created using the 4-layer Finite Element Model (FEM) as implemented in BESA v7.0. The warm colors (red to yellow) on the map reflect greater activation during delay period while the cold colors (blue) reflect greater activation during encoding period.

Classification Based on fMRI-Weighted Thalamic Source Amplitude and Task Performance

To understand the relationship between thalamic sources and task performance, classification was performed in Matlab (R2022b) using linear discriminant analysis. This was done using the measurements of fMRI-weighted left thalamus mean source amplitudes between 160 and 390 ms during the delay period and percent correct task performance (the predictors) and the known class labels (low or high load condition). The *fitcdiscr* function was used to perform linear discriminant analysis (LDA) on training data (observations with the known class labels). Then the *resubLoss* function was used to compute the resubstitution error, which is the misclassification error (or proportion of misclassified observations) on the training set. Next the confusion matrix was

computed on the training set using the *confusionchart* function from the output of the *resubPredict* function. The number and percentage of observations that were misclassified by the linear discriminant function were computed and visualized by graphing the correctly and misclassified data points as a function of working memory load. The linear discriminant function separated a plane defined by thalamic source amplitude and task performance into regions defined by working memory load. These regions were visualized by creating a grid of values and applying the classification function to that grid.

Since the resubstitution error can under-estimate the test error (generalization error), which is the expected prediction error on an independent dataset, we conducted cross-validation by randomly dividing the data. We used the *cvpartition* function to generate 10 disjoint stratified subsets, and then used the *crossval* and *kfoldLoss* functions to estimate the true test error for LDA using 10-fold stratified cross-validation.

Results

Pilot Experiment Behavioral Results

In the pilot behavioral experiment, an effect of load was found for both the WM task and immediate recognition task. For the long-term recognition task, there was a significant interaction between load and the delay period condition, with performance that was worse for the high-load condition when the scrambled images were presented during the delay relative to simple fixation during the delay (*Supplementary Figure 6).*

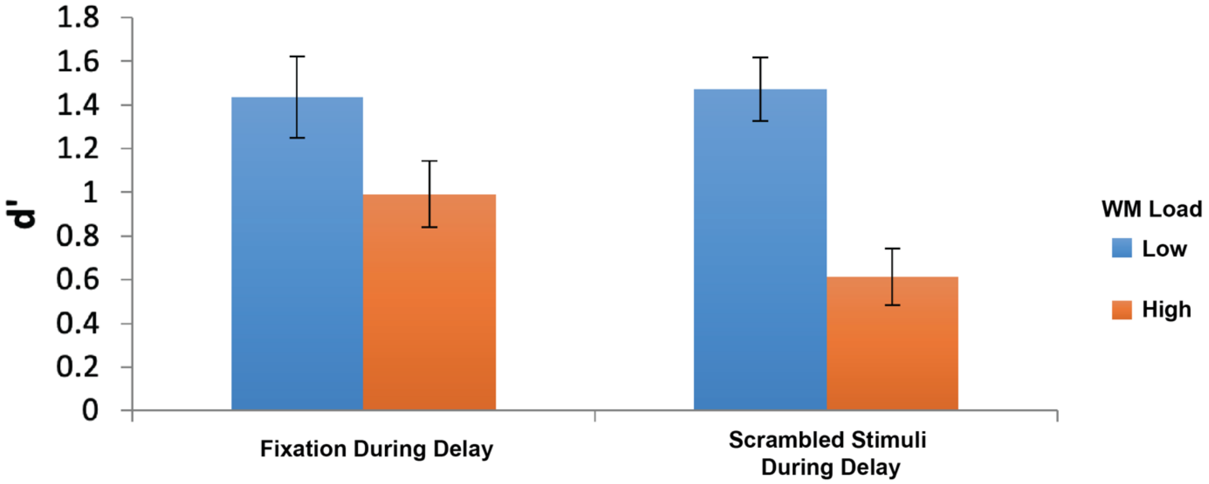

Supplementary Figure 6. Behavioral Differences as a Function of Delay Period Content. D-prime sensitivity was better when the delay period contained a simple fixation condition compared to scrambled stimuli. These findings directly motivated the present main EEG study because it suggested that the presentation of phase-scrambled scenes during the delay period induced more interference during maintenance as evidenced by reduced performance on the subsequent memory task.

fMRI-Weighted EEG Source Analysis

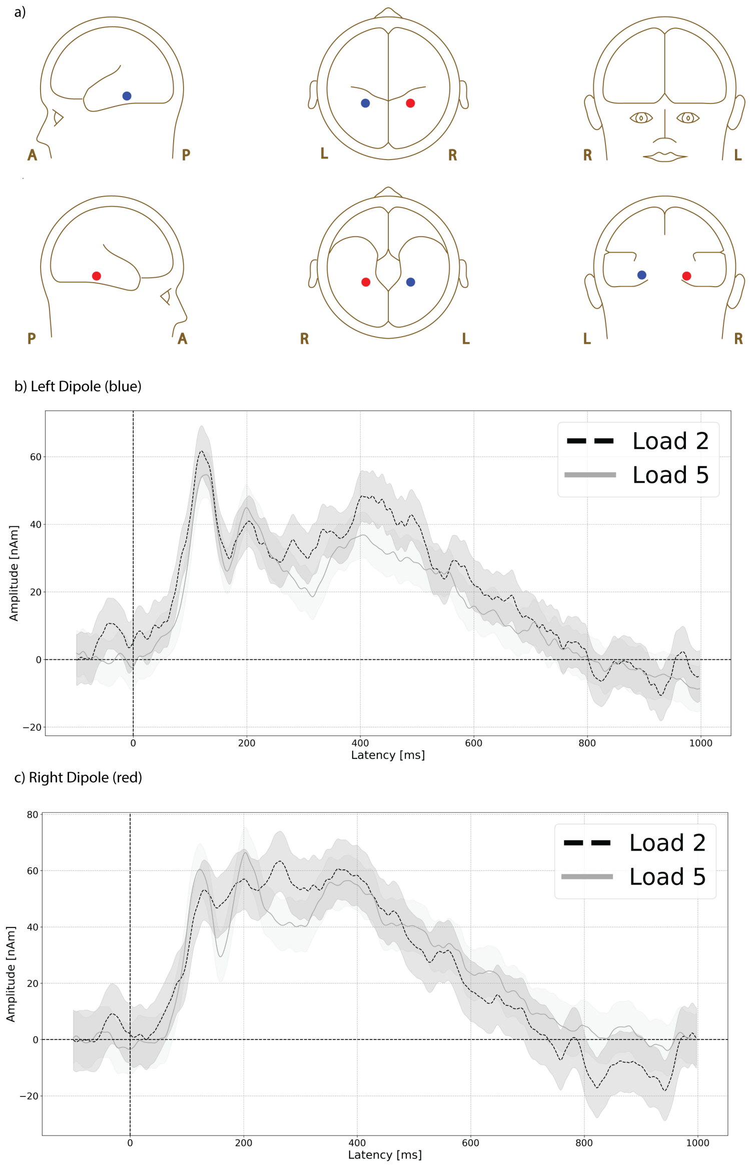

Supplementary Figure 7. Group Source Waveform during Encoding a) Head plot of asymmetric dipole clusters located in parahippocampal gyrus in left (blue) and right (red) hemispheres. [Cluster 1 (blue): p = 0.486]. b) Source Analysis results for high (Load 5)- and low (Load 2)-load conditions for 1-dipole (left hemisphere) solution during encoding (Baseline: -100 msec, Encoding Period: 0 to 1000 msec of 1400 msec period). C) Source Analysis results for high (Load 5)- and low (Load 2)-load conditions for 1-dipole (left hemisphere) solution during encoding (Baseline: -100 msec, Encoding Period: 0 to 1000 msec of 1400 msec period). Group source-derived waveforms (grand average) of Load 2 (black) and Load 5 (gray) in parahippocampal gyrus do not show a significant WM load effect [p = 0.486] at any time period in left or right hemisphere.

fMRI-Weighted Thalamic Source Amplitude and Task Performance Classification

We estimated the true test error for linear discriminant analysis by using the *cvpartition* function to generate 10 disjoint stratified subsets, followed by the *crossval* and *kfoldLoss* functions to compute a 10-fold stratified cross-validation. Over the 38 training observations, classification accuracy for the load condition was 73.68% with 26.32% (*ldaCVErr*=0.2632) of subject datapoints misclassified by the linear discriminant function.

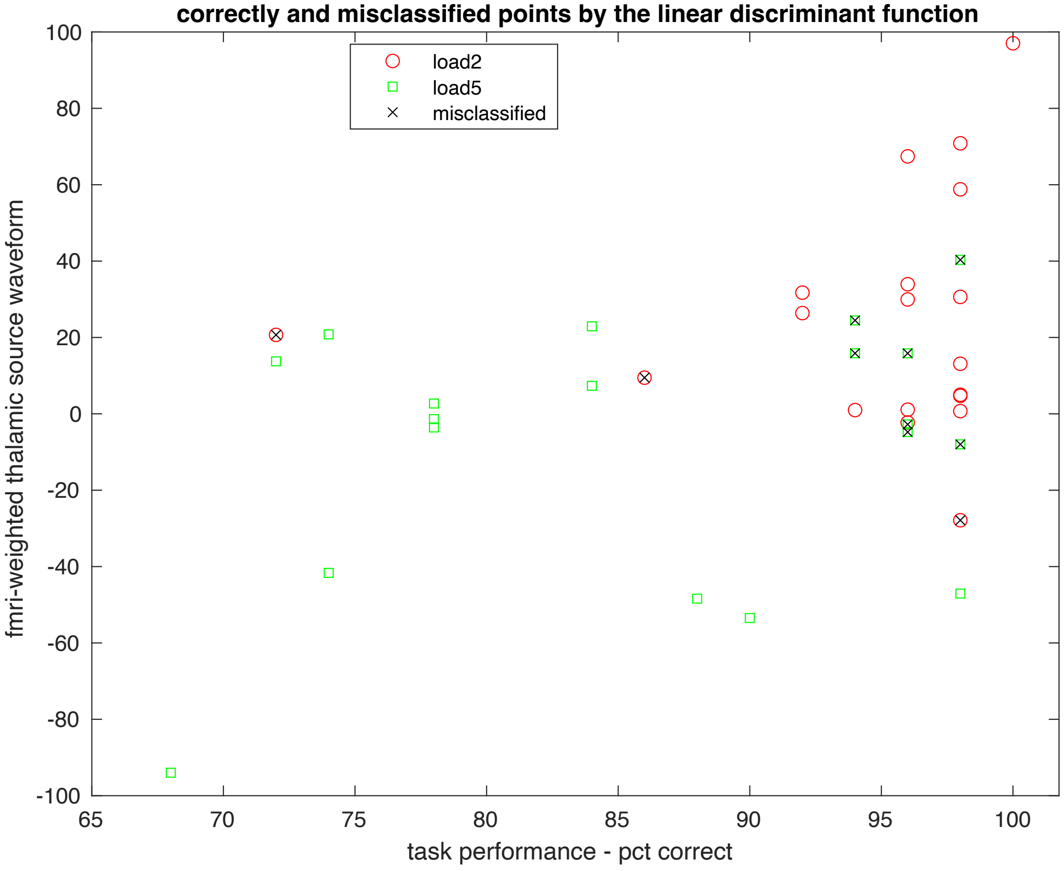

Supplementary Figure 8. Linear Discriminant Function Classification. The graph displays correctly classified and misclassified subjects based on their working memory load using a linear discriminant function. The predictors are the fMRI-weighted left thalamic source waveform mean amplitudes between 160 and 390 ms into the working memory delay period (y-axis) and percent correct task performance (x-axis).

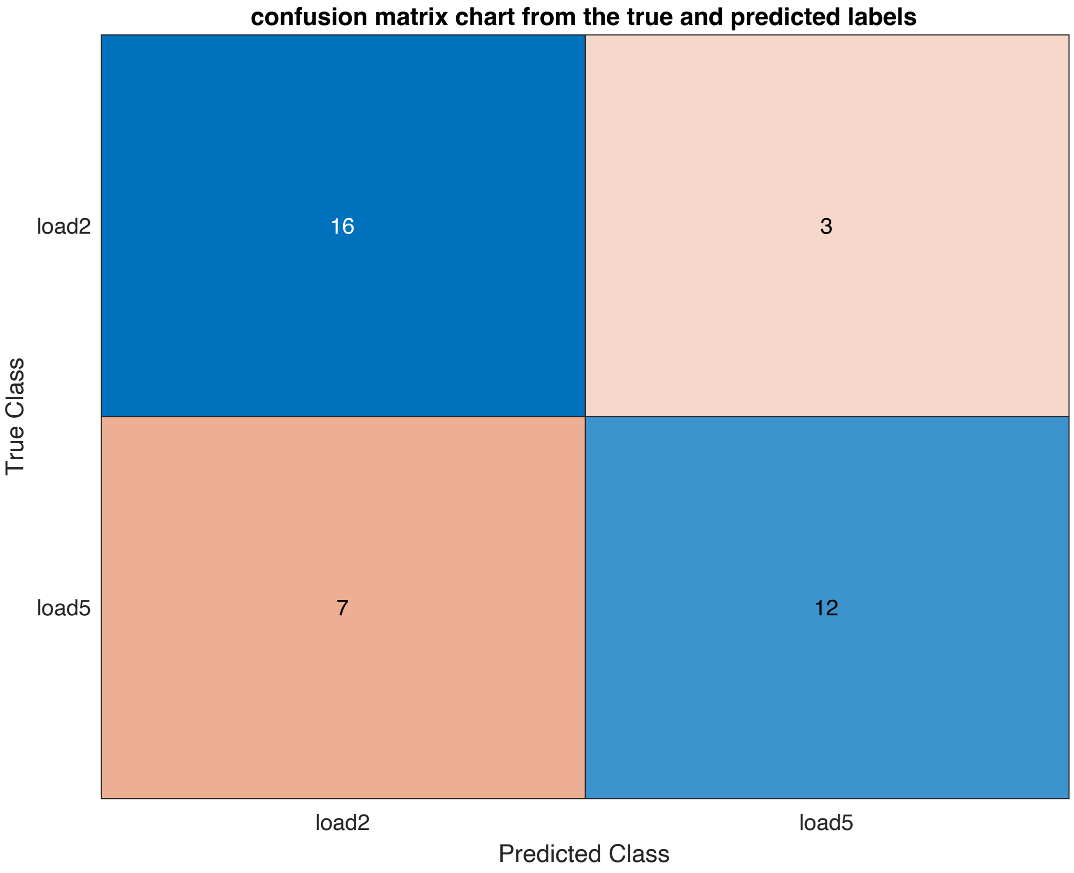

Supplementary Figure 9. Linear Discriminant Analysis Confusion Matrix. The graph displays the confusion matrix. The (i,j) element in the matrix is the number of samples whose known class label is class i (y-axis) and whose predicted class is j (x-axis). The diagonal elements represent correctly classified observations (28 out of 38, or 73.68%).

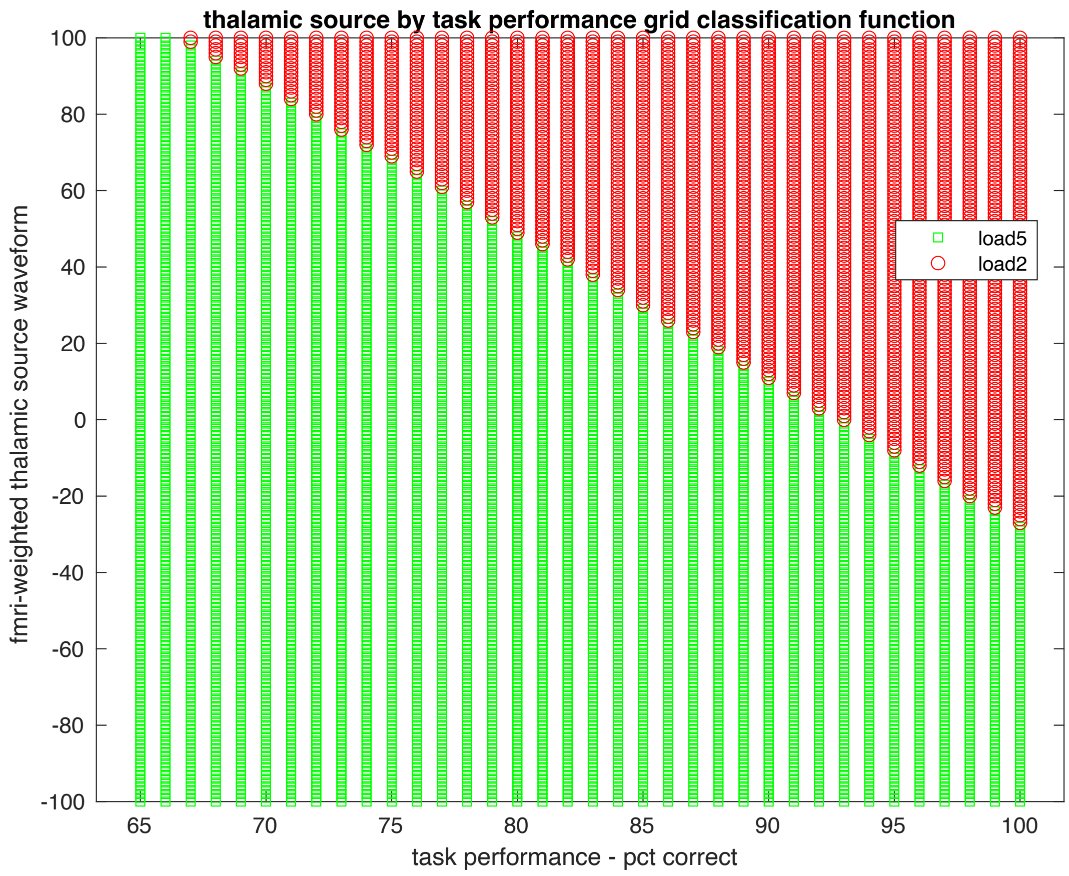

Supplementary Figure 10. Linear Discriminant Analysis Classification Function Grid. The LDA function separated the plane into regions divided by a line and assigned different regions to different working memory loads. To visualize these two regions, a grid of values (*x – thalamus source activity, y – task percent correct*) was created and the classification function was applied to that grid.
